# Post-cholecystectomy clip migration—a unique method of retrieval

**DOI:** 10.1093/jscr/rjab640

**Published:** 2022-06-30

**Authors:** Ezekiel Aaron, Pranavan Palamuthusingam

**Affiliations:** James Cook University, Townsville, Australia; Townsville University Hospital, Townsville, Australia

## Abstract

Post-cholecystectomy clip migration with formation of bile duct stones is a known but rare complication of laparoscopic cholecystectomy. This report discusses the case of a 64-year-old lady who presented with biliary colic symptoms 12 years post laparoscopic cholecystectomy. Computed tomography of the abdomen demonstrated one surgical clip located in the distal common bile duct, with a bile stone formed around it. This was removed via ERCP with spyglass cholangioscopy and lithotripsy.

## INTRODUCTION

Laparoscopic cholecystectomy is the mainstay for treatment of gallstone disease. It is a safe operation with a similar rate of complications to the open approach [1]. Complications include bile duct damage, bile duct leakage, infection, strictures and post-cholecystectomy clip migration (PCCM; [2]). PCCM is a rare complication that occurs when the surgical clip used to close the cystic duct migrates into the common bile duct (CBD; [3–5]). The majority of cases are treated successfully with ERCP but ~20% of cases require an alternative approach [3, 4]. The following case required a total of three endoscopic retrograde cholangiopancreatographies (ERCPs), the third using spyglass cholangioscopy and lithotripsy in order to extract the surgical clips and gallstone.

## CASE REPORT

A 64-year-old woman presented with several months of epigastric pain, nausea and vomiting exacerbated by fatty meals. Past surgical history included a laparoscopic cholecystectomy 12 years ago and a hysterectomy for cervical cancer 30 years ago.

**Figure 1:**
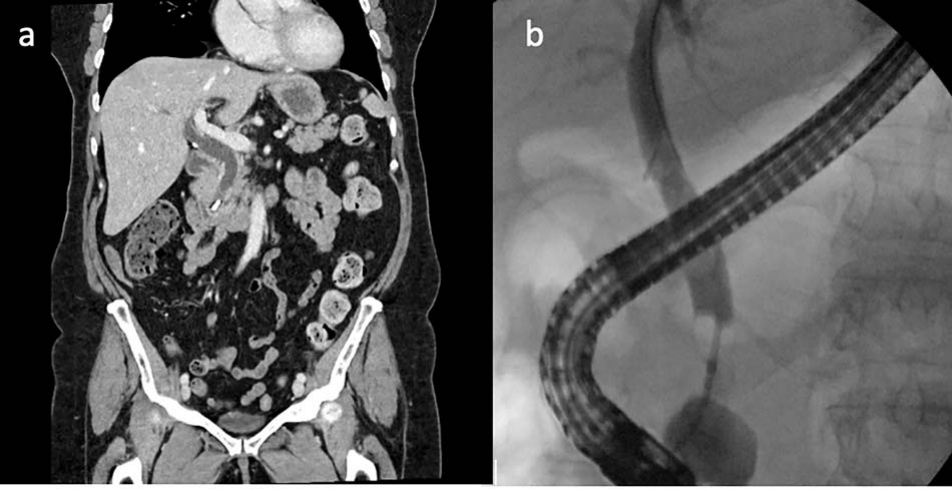
CT abdomen and ERCP. (**a**) CT abdomen showed a linear high density focus consistent with a 10-mm surgical clip obstructing the distal CBD, which was significantly dilated proximally (coronal). (**b**) ERCP showing filling defect in CBD.

On examination, she was febrile at 38.1, abdomen was soft with epigastric tenderness and negative murphy’s sign. Laboratory investigations showed markedly deranged LFTs indicating a cholestasis pattern: aspartate aminotransferase (AST)—63 (10–35), alanine aminotransferase (ALT)—102 (5–30), alkaline phosphatase (ALP)—872 (30–115) and GGT—987 (5–35). Computed tomography (CT) abdomen showed a linear high density focus consistent with a 10-mm surgical clip obstructing the distal CBD, which was significantly dilated proximally (Fig. 1) two attempts were made to remove the surgical clips via ERCP and balloon extraction however the clip and stone remained impacted above the ampulla, which was quite edematous. After each unsuccessful attempt the bile duct was re-stented to allow biliary drainage and she was placed on antibiotic therapy to reduce the infection and resultant inflammation.

**Figure 2:**
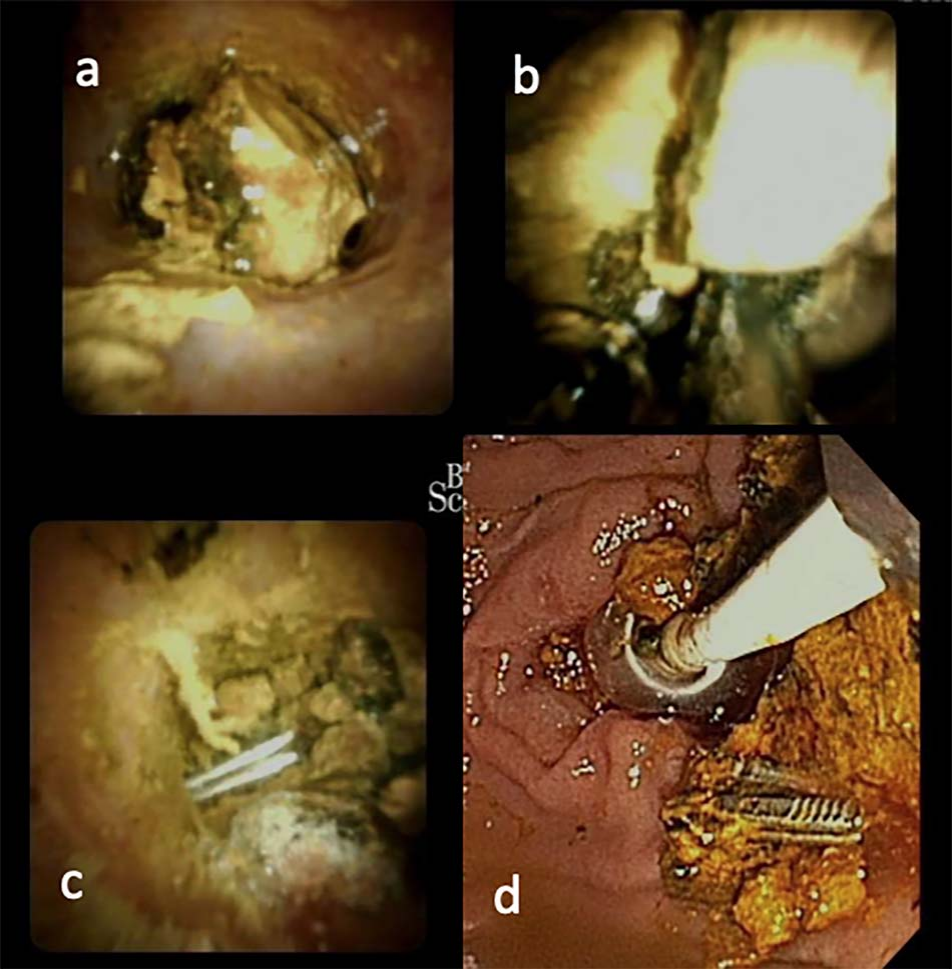
ERCP with spyglass cholangioscopy. (**a**) Bile duct stone obstructing CBD. (**b**) EHL fragmentation of stone. (**c**) Two Surgical clips freed from stone material. (**d**) Biliary tree swept with balloon and basket.

She was then referred to a larger tertiary center for spyglass cholangioscopy that enabled the surgeon to directly visualize the pathology within the bile duct. They found two surgical clips with a surrounding 30-mm stone impacted in the distal CBD. Electrohydraulic lithotripsy was used to divide the stone into smaller fragments, which were retrieved via balloon/basket extraction (Fig. 2). She was then admitted for 5 days and discharged once her liver function tests (LFTs) had stabilized.

## DISCUSSION

Around 100 cases of PCCM have been reported in the literature. The majority of cases presented 26 months post-cholecystectomy with symptoms characteristic of biliary colic. Most cases were diagnosed with abdominal ultrasound and successfully treated by ERCP. Small percentage of cases required surgical exploration of the bile duct or percutaneous transhepatic drainage [3–6].

Several theories have been put forward seeking to explain the pathogenesis of PCCM. Kitamura *et al*. suggested that pressure exerted by the liver leads to inversion of the cystic duct stump into the CBD. The stump undergoes necrosis over time, which allows the surgical clips to migrate further down the CBD [7].

Another theory suggests that incorrect surgical clip placement results in bile duct leakage and biloma formation. The resultant inflammation surrounding the area leads to erosion of the cystic stump and CBD wall allowing the surgical clips to migrate into the CBD. Then the surgical clip acts as a nidus for the bile to aggregate around and form a stone, which obstructs the bile duct [4, 8].

Although there are no known deaths due to PCCM, severe complications can result from clip migration such as cholangitis and pancreatitis [3, 8–10]. To prevent this from occurring careful effort should be made when placing surgical clips on the cystic duct and the number of clips should be limited [3, 4]. Absorbable sutures are another alternative to metal clips however they can also become nidus for gallstone formation [11, 12].

This case shows the possibility of using lithotripsy with spyglass cholangioscopy as a potentially safer, non-invasive alternative to surgery in the treatment of refractory bile duct stones [13, 14]. Furthermore, Spyglass cholangioscopy enables the endoscopist to directly visualize the bile duct for accurate diagnosis and treatment of pathology [13, 15]. However, equipment availability and training requirements make its widespread use less feasible.

## CONCLUSION

In conclusion, PCCM should be considered in the differentials list for patients who present any time period post-cholecystectomy with obstructive jaundice symptoms. This case highlights the utility of using ERCP with spyglass and lithotripsy to extract difficult bile duct stones. These newer technologies are more expensive, poorly accessible and more research is required before recommending their wider use.

## CONFLICT OF INTEREST STATEMENT

None declared.

## FUNDING

This research did not receive any specific grant from funding agencies in the public, commercial or not for profit sectors.
